# 

**Published:** 1891-05

**Authors:** 


					﻿Vol. XI.
MAY, 1891.
NO. 5.
IBHE
pOMfEOPATHlC
A Monthly Journal of Homoeopathic Materia Medica
and Clinical Medicine.
“ He is a freeman whom truth makes free, and all are slaves beside."
"Seek the Truth: come whence it may, cost what it will.”
EDITED AND PUBLISHED BY
WALTER ML JAMES, M. D.,
AND
George H. Clark, m. d.
PHILADELPHIA:
No. 1125 SPRUCE STREET.
LONDON"
ALFRED HEATH & CO., Sole Agents for Great Britain,
114 Ebury Street, Eaton Square.
■SS'l'eariy Subscription, $2.SO, invariably in advance. Single numbers, 2S cts.
Entered at the Philadelphia Paet-Office as Second-Class Mail Matter.
				

